# Genome‐Wide Identification and Stress‐Responsive Expression of the *JAZ* Gene Family in *Cinnamomum burmannii*

**DOI:** 10.1002/pld3.70195

**Published:** 2026-10-21

**Authors:** Wei Lin, Xuemei Chen, Yuqi Li, Xiaojin Liu, Zhiyi Cui

**Affiliations:** ^1^ Research Institute of Tropical Forestry Chinese Academy of Forestry Guangzhou China; ^2^ Foshan Institute of Forestry (Foshan Botanical Garden) Foshan China

**Keywords:** *Cinnamomum burmannii*, drought stress, expression analysis, hormone response, *JAZ* gene family

## Abstract

JASMONATE ZIM‐DOMAIN (JAZ) proteins act as central repressors of jasmonate signaling, modulating plant development, stress resilience, and secondary metabolism. Despite the economic and ecological importance of the aromatic tree *Cinnamomum burmannii* , its *JAZ* family has not been systematically explored. We performed a genome‐wide survey and identified 12 *CbJAZ* genes, all harboring conserved TIFY and Jas domains and unevenly distributed across seven chromosomes. Phylogenetic reconstruction assigned them to four clades, each exhibiting conserved exon–intron structures and motif compositions. Segmental duplication events primarily drove the expansion of this family. Promoter *cis*‐element analysis revealed a rich repertoire of hormone‐, light‐, and stress‐responsive motifs. Tissue‐specific expression profiling showed that most *CbJAZ* transcripts accumulate preferentially in flowers and fruits. Upon exogenous methyl jasmonate (MeJA) treatment, the majority of *CbJAZ* genes were significantly induced, whereas abscisic acid (ABA) and drought stress generally suppressed their expression. Notably, *CbJAZ8* and *CbJAZ9* emerged as robust responders to all three treatments, and their encoded proteins localized to the nucleus, implicating them as candidate genes for future investigation of JA, ABA, and drought signaling crosstalk and potential regulators of essential oil biosynthesis. This first comprehensive characterization of the *CbJAZ* family not only furnishes a valuable genetic resource but also pinpoints *CbJAZ8* and *CbJAZ9* as prime candidates for future investigations into JA‐mediated stress adaptation and essential oil regulation in this economically relevant tree species.

## 1 Introduction

*Cinnamomum burmannii* (Nees & T. Nees) Blume, a member of the Lauraceae family, is an evergreen tree native to southeastern China, Indonesia, and the Philippines (Al‐Dhubiab 2012). Its bark, leaves, and roots are rich in essential oils containing diverse terpenoid volatile compounds. Five chemotypes of *C. burmannii* have been recognized based on the chemical polymorphism of its major foliar terpenes: camphor‐type, alpha‐terpineol‐type, borneol‐type, terpinen‐4‐ol‐type, and 1,8‐cineole‐type (Hou et al. 2023). Notably, D‐borneol, the signature component, exhibits anti‐inflammatory (Zhang et al. 2025), antimicrobial (Shi, Lin, et al. 2024), and transdermal drug‐enhancing properties (Zhang, Lin, et al. 2024), making it widely used in pharmaceuticals, fragrances, and cosmetics (Zhang, Cai, et al. 2026). However, the natural essential oil content in *C. burmannii* is relatively low under conventional cultivation, and the regulatory mechanisms governing essential oil biosynthesis remain largely unexplored. Therefore, understanding the molecular components of JA signaling, especially the JAZ repressors, is a prerequisite for developing targeted strategies to improve essential oil yield.

As lipid‐derived phytohormones, jasmonates (JA and MeJA) serve as central regulators of diverse plant processes, including growth, development, and stress resistance to both biotic and abiotic challenges (Howe et al. 2018). Jasmonates are ubiquitous signaling molecules in land plants (Chini et al. 2023), and the jasmonate perception and signaling pathway is well‐conserved across land plant lineages (Bowman et al. 2017; Gasperini and Howe 2024; Liang et al. 2024). The core mechanism of the JA signaling pathway relies on the JASMONATE ZIM‐DOMAIN (JAZ) protein family, which functions as a central negative regulator (Thines et al. 2007; Chini et al. 2007). JAZ proteins act as repressors by physically interacting with MYC2 and other transcription factors, which prevents the expression of JA‐responsive genes under resting conditions (Zhang, Xu, et al. 2026). When plants are challenged by stress, bioactive JA‐Ile accumulates and promotes the association of the SCFCOI E3 ubiquitin ligase complex with JAZ proteins, marking them for ubiquitination and 26S proteasome‐mediated degradation (Sheard et al. 2010; Fonseca et al. 2009). As a result, downstream transcription factors are released from repression, enabling the expression of JA‐responsive genes that function in stress defense and secondary metabolism (Zhang et al. 2015; Fernández‐Calvo et al. 2011; Qi et al. 2015; Chini et al. 2016). In addition to the canonical COI1‐dependent pathway, emerging evidence suggests that alternative COI1‐independent degradation mechanisms may also operate in certain plant species, adding another layer of regulatory complexity (Srivastava et al. 2018).

JAZ repressors are defined by two signature domains: the N‐terminal TIFY motif (alternatively named ZIM), which not only facilitates both homotypic and heterotypic interactions among JAZ proteins (Chung and Howe 2009; Chini et al. 2009) but also recruits the co‐repressor NINJA and TOPLESS (TPL) proteins, forming a repression complex that actively silences JA‐responsive genes (Pauwels et al. 2010). The ability of JAZ proteins to interact with COI1 and MYC2 depends on the Jas domain (CCT_2 motif) at the C‐terminus (Sheard et al. 2010). A conserved degron sequence required for COI1‐mediated ubiquitination is embedded within the Jas domain, and mutations in this degron sequence render JAZ proteins resistant to degradation, leading to constitutive repression of JA responses (Zhou et al. 2025). Beyond their core function in JA signaling, JAZ proteins have been shown to integrate crosstalk with other phytohormonal pathways (Fonseca et al. 2018), including abscisic acid (ABA) (Lin et al. 2025), ethylene (ETH) (Liu et al. 2022), and auxin (IAA) (Mei et al. 2023), thereby orchestrating complex regulatory networks that fine‐tune plant growth and defense trade‐offs.

So far, the *JAZ* gene family has been systematically characterized in numerous plant species, including *Arabidopsis thaliana* (13 members) (Liu et al. 2021), *Populus trichocarpa* (22 members) (Yang et al. 2023), *Oryza sativa* (15 members) (Ye et al. 2009), *Solanum lycopersicum* (12 members) (Chini et al. 2017), and *Triticum durum* (54 members) (Ebel et al. 2018). These studies have revealed that the number of *JAZ* genes varies considerably among species, largely due to lineage‐specific gene duplication events, yet their core domains and regulatory functions remain highly conserved. Functional analyses have demonstrated that *JAZ* genes are involved not only in biotic stress responses (Haq et al. 2025) but also in tolerance to abiotic stresses such as salinity (Zhao et al. 2025), drought (Xu and Wang 2026), and extreme temperatures (Du et al. 2025). Notably, the JAZ‐MYC2 regulatory module has emerged as a conserved hub linking JA signaling to secondary metabolism. For example, in *Ginkgo biloba* , GbJAZ proteins sequester GbMYC2 to repress terpenoid trilactone biosynthesis under nonstressed conditions, and MeJA treatment triggers JAZ degradation, thereby releasing GbMYC2 to activate downstream terpene synthase genes (Zheng et al. 2024). Similarly, in *Salvia miltiorrhiza* , SmJAZ3 negatively regulates tanshinone accumulation through interaction with SmMYC2 (Cao et al. 2024), whereas in *Curcuma wenyujin*, CwJAZ directly binds CwMYC2 to inhibit terpenoid synthesis (Wang et al. 2024). In *Camellia sinensis* , CsJAZ1 and CsJAZ2 have been shown to regulate flavonoid biosynthesis by interacting with CsMYC2, and their expression is modulated by both JA and environmental cues (Zhu et al. 2022). These findings collectively point to the *JAZ* family as a key nexus linking environmental signals to secondary metabolic outputs in diverse plants.

Despite these advances in other plant species, the *JAZ* gene family in *C. burmannii* has not been systematically identified, and the role of JAZ proteins in regulating terpenoid biosynthesis in this aromatic tree remains unexplored. A genome‐wide investigation of the *JAZ* family in *C. burmannii* was undertaken here for the first time to bridge this gap. Additionally, we analyzed their expression profiles across different tissues and under MeJA, ABA, and drought treatments, along with their sequence features, evolutionary relationships, and *cis*‐regulatory elements. Our results will provide candidate genes for elucidating JA‐mediated stress responses and terpenoid regulation in *C. burmannii* .

## 2 Materials and Methods

### 2.1 Plant Materials and Treatment

Three‐year‐old clonal *C. burmannii* plants growing at Foshan Botanical Garden, Foshan, China, were used as plant materials in this study. Different tissue samples, including roots, young stems, leaves (at four developmental stages designated S1–S4), flowers, and fruits, were collected from healthy plants for tissue‐specific expression analysis. For hormonal and drought stress treatments, healthy and uniform seedlings were subjected to three independent treatments: (1) 100‐μM methyl jasmonate (MeJA) (Sigma‐Aldrich); (2) 100‐μM ABA (Sigma‐Aldrich); and (3) 20% (w/v) polyethylene glycol (PEG) 6000 (Sinopharm, Beijing, China) to simulate drought stress. Leaf tissues were collected immediately before treatment (0 h) as the baseline, and again at 6, 12, and 24 h after treatment initiation. All specimens were immediately frozen in liquid nitrogen and kept at −80°C for subsequent analysis.

### 2.2 Identification of *JAZ* Family in *C. burmannii*

The genome assembly and annotation files of *C. burmannii* were sourced from a previously published study (Hou et al. 2025), and JAZ protein sequences from *A. thaliana* were downloaded from the TAIR database (https://www.arabidopsis.org/) and used as queries for BLAST searches. Two methods were used to identify members of the *JAZ* gene family in *C. burmannii* : (1) bidirectional blastp alignment in TBtools (Chen et al. 2020), with an E‐value of 1e‐5, was used to screen potential *JAZ* family genes in the *C. burmannii* genome; (2) the Hidden Markov Model (HMM) profiles of JAZ's conserved domains TIFY (PF06200) and CCT_2 (PF09425) were downloaded from the Pfam database, and an HMMER search was performed using the hmmsearch program in HMMER 3.0 to extract protein sequences containing the corresponding domains. The results obtained from the two methods were combined and submitted to the NCBI Conserved Domain Database (CDD) and InterPro (https://www.ebi.ac.uk/interpro/) for conserved domain verification (*E*‐value ≤ 1e‐10). Sequences with conserved domain deletions or incompleteness were removed to obtain the final set of JAZ genes in the *C. burmannii* genome. Physicochemical properties such as protein length, isoelectric point, grand average of hydropathicity (GRAVY), and instability index were analyzed using the ExPASy ProtParam tool (https://web.expasy.org/protparam/). Subcellular localization prediction for all CbJAZ proteins was performed using WoLF PSORT (https://wolfpsort.hgc.jp/).

### 2.3 Phylogeny, Gene Structure, and Conserved Motif Analysis

Multiple sequence alignments of the CbJAZ protein sequences were performed using MAFFT v7.0 with default parameters and visualized using Jalview v2.11. To reconstruct phylogenetic relationships, JAZ protein sequences from *A. thaliana* , *P. trichocarpa* , and *O. sativa* were retrieved from the NCBI database. Phylogenetic trees were constructed using both neighbor‐joining (NJ) and maximum likelihood (ML) methods in MEGA v7.0. For the NJ analysis, the Poisson substitution model was used with pairwise deletion of gaps and 1000 bootstrap replicates. For the ML analysis, the best‐fit amino acid substitution model determined by MEGA's model selection tool was applied, also with 1000 bootstrap replicates. Trees were visualized using iTOL (https://itol.embl.de/), and the NJ tree was selected as the main figure for presentation. Gene structures were determined by aligning coding sequences (CDSs) to genomic sequences using TBtools v1.098. Conserved motifs were identified using the MEME Suite (https://meme‐suite.org/meme/) with a maximum of 10 motifs, motif width of 6–50 amino acids, and any number of repetitions. The resulting motifs, gene structures, and conserved domains were integrated and visualized using TBtools.

### 2.4 Chromosomal Mapping and Promoter *cis*‐Acting Element Analysis

Gene density analysis was performed on genome files using TBtools and applied to chromosomes. At the same time, the corresponding positions of *CbJAZ* genes were determined based on annotation files. The chromosomal distribution of all *CbJAZ* genes was determined, and their promoter regions were analyzed using the 2 kb upstream sequences extracted via TBtools, submitted them to the PlantCARE website (http://bioinformatics.psb.ugent.be/webtools/plantcare/), search for all *cis*‐acting elements in the promoter region, screen, analyze and remove redundancies, functionally relevant *cis*‐acting elements were selected, and then visualize them using TBtools. Each *cis*‐acting element in the figure is represented by different colors, and their positions represent their length in the promoter region.

### 2.5 RNA Extraction and qRT‐PCR Analysis

Total RNA was isolated from all specimens using TRIzol reagent (Invitrogen, MA, USA) following the supplier's protocol. RNA quality was evaluated via 1% agarose gel electrophoresis and with the RNA Nano 6000 Assay Kit on a Bioanalyzer 2100 system (Agilent Technologies, CA, USA). RNA purity was determined with a NanoPhotometer spectrophotometer (IMPLEN, CA, USA), and concentration was measured using a Qubit 2.0 Fluorometer (Life Technologies, CA, USA). First‐strand cDNA was synthesized from 1 μg of total RNA using Hifair III 1st Strand cDNA Synthesis SuperMix (gDNA digester plus) (YEASEN, Shanghai, China). qRT‐PCR was performed on an FQD‐96A instrument (BIOER, Hangzhou, China) with RPS15 as the internal reference gene (Shi, Cai, et al. 2024), and relative expression levels were calculated using the 2^−ΔΔCt^ method with three biological replicates for each sample. Primers are listed in Table S1. To visualize tissue‐specific expression, heatmaps were generated from the qRT‐PCR data using TBtools, with clustering based on Euclidean distance and Ward's method. For hormone and stress treatments, data were analyzed and plotted as bar charts or line graphs using GraphPad Prism 10 (version 10.3.0).

### 2.6 Subcellular Localization Analysis

To determine the subcellular localization of CbJAZ8 and CbJAZ9, their ORFs were amplified with specific primers (Table S1), and the resulting amplicons were used for the generation of CbJAZ8‐GFP and CbJAZ9‐GFP fusion constructs in the vector PC2300sGFP. The recombinant constructs and the empty vector were transformed into *Agrobacterium* GV3101 and infiltrated into tobacco leaves. After 48 h of dark incubation, GFP fluorescence, mCherry signals, and chlorophyll autofluorescence were captured by confocal microscopy. Co‐localization with chloroplasts was evaluated by merging the fluorescent channels with bright‐field images.

### 2.7 Essential Oil Extraction and Content Determination

Fresh leaf samples were ground into fine powder in liquid nitrogen. The powder (10 g, fresh weight) was subjected to hydrodistillation for 3 h using a Clevenger‐type apparatus with an appropriate volume of distilled water. The mixture was kept at continuous boiling throughout the distillation. After distillation, the essential oil volume was recorded from the graduated collection tube. Oil yield (v/w %) was calculated as (volume of essential oil/fresh weight of sample) × 100. Three independent biological replicates were performed for each sampling time (0, 6, 12, and 24 h) after MeJA treatment.

### 2.8 Statistical Analysis

All experiments were performed with at least three independent biological replicates. Data are expressed as mean ± standard deviation (SD). For qRT‐PCR time‐course data under MeJA, ABA, and drought treatments, statistical comparisons between each treatment time point and the corresponding control were performed using one‐way ANOVA followed by Dunnett's multiple comparison test (*p* < 0.05). Pearson correlation coefficients between *CbJAZ* expression levels and essential oil content were also calculated, with statistical significance set at *p* < 0.05. All statistical analyses and graphical representations were performed using GraphPad Prism v10.3.0 (GraphPad Software, Boston, MA, USA).

## 3 Results

### 3.1 Genome‐Wide Identification of the *CbJAZs* Family

After removing sequences that lacked either the TIFY domain or the Jas domain, as well as redundant sequences, 12 *JAZ* gene family members, designated *CbJAZ1* to *CbJAZ12*, were identified based on their chromosomal positions. All 12 proteins contained the conserved TIFY and Jas domains. The *CbJAZ* genes encode proteins ranging from 117 to 392 amino acids, with CDS lengths spanning 354–1179 bp (Table 1). The molecular weights ranged from 13.08 to 41.67 kDa, and the isoelectric points (pI) were between 8.78 and 9.91. Notably, the pI values of all CbJAZ proteins were greater than 7, indicating that they are basic proteins. Among them, CbJAZ7 had the longest protein length and the highest molecular weight. The instability index of every family member exceeded 40, suggesting that all CbJAZ proteins are predicted to be unstable in vitro, and all 12 CbJAZ proteins exhibited negative GRAVY values, indicating their hydrophilic character. WoLF PSORT‐based subcellular prediction indicated nuclear localization for all CbJAZ proteins.

**TABLE 1:** Molecular characteristics of *CbJAZ* genes.

Gene name	Gene ID	Protein length (aa)	CDS length (bp)	pI	Instability index	Molecular weight (kDa)	GRAVY	Predicted subcellular location
CbJAZ1	Cbur01G011770	264	795	8.82	44.39	28.86	−0.615	Nucleus
CbJAZ2	Cbur01G013000	165	498	9.26	72.02	17.23	−0.316	Nucleus
CbJAZ3	Cbur01G020340	255	768	9.37	49.68	27.88	−0.715	Nucleus
CbJAZ4	Cbur02G002180	304	915	9.91	40.14	32.51	−0.332	Nucleus
CbJAZ5	Cbur02G034170	391	1176	9.25	48.09	40.93	−0.257	Nucleus
CbJAZ6	Cbur03G009160	355	1068	8.87	68.00	37.94	−0.823	Nucleus
CbJAZ7	Cbur04G003790	392	1179	9.57	43.08	41.67	−0.322	Nucleus
CbJAZ8	Cbur07G017660	261	786	9.84	49.32	28.75	−0.425	Nucleus
CbJAZ9	Cbur10G002720	148	447	9.32	80.14	16.45	−0.709	Nucleus
CbJAZ10	Cbur12G015640	117	354	9.46	69.68	13.08	−0.687	Nucleus
CbJAZ11	Cbur12G016400	219	660	9.54	54.06	24.21	−0.421	Nucleus
CbJAZ12	Cbur12G018060	123	372	8.78	98.09	13.75	−0.667	Nucleus

### 3.2 Multiple Sequence Alignment and Phylogenetic Analysis

Both the central TIFY domain and the C‐terminal Jas domain were highly conserved, as revealed by CbJAZ protein sequence alignment (Figure 1). A phylogenetic tree was constructed using the NJ method with 1000 bootstrap replicates to infer the evolutionary relationships of JAZ proteins from *C. burmannii* , *P. trichocarpa* , *A. thaliana* , and *O. sativa* . The 12 CbJAZ proteins were phylogenetically classified into four subgroups (Figure 2), following the grouping system of *Arabidopsis* JAZs. The largest subgroup (Group IV) included five members, whereas the smallest (Group II) consisted solely of CbJAZ10. Groups I and III both contained three members each. Notably, most CbJAZ proteins were more closely related to JAZ sequences from the woody perennial *P. trichocarpa* than to those from *O. sativa* or *A. thaliana* , reflecting their shared evolutionary history as dicotyledonous trees.

**FIGURE 1:**
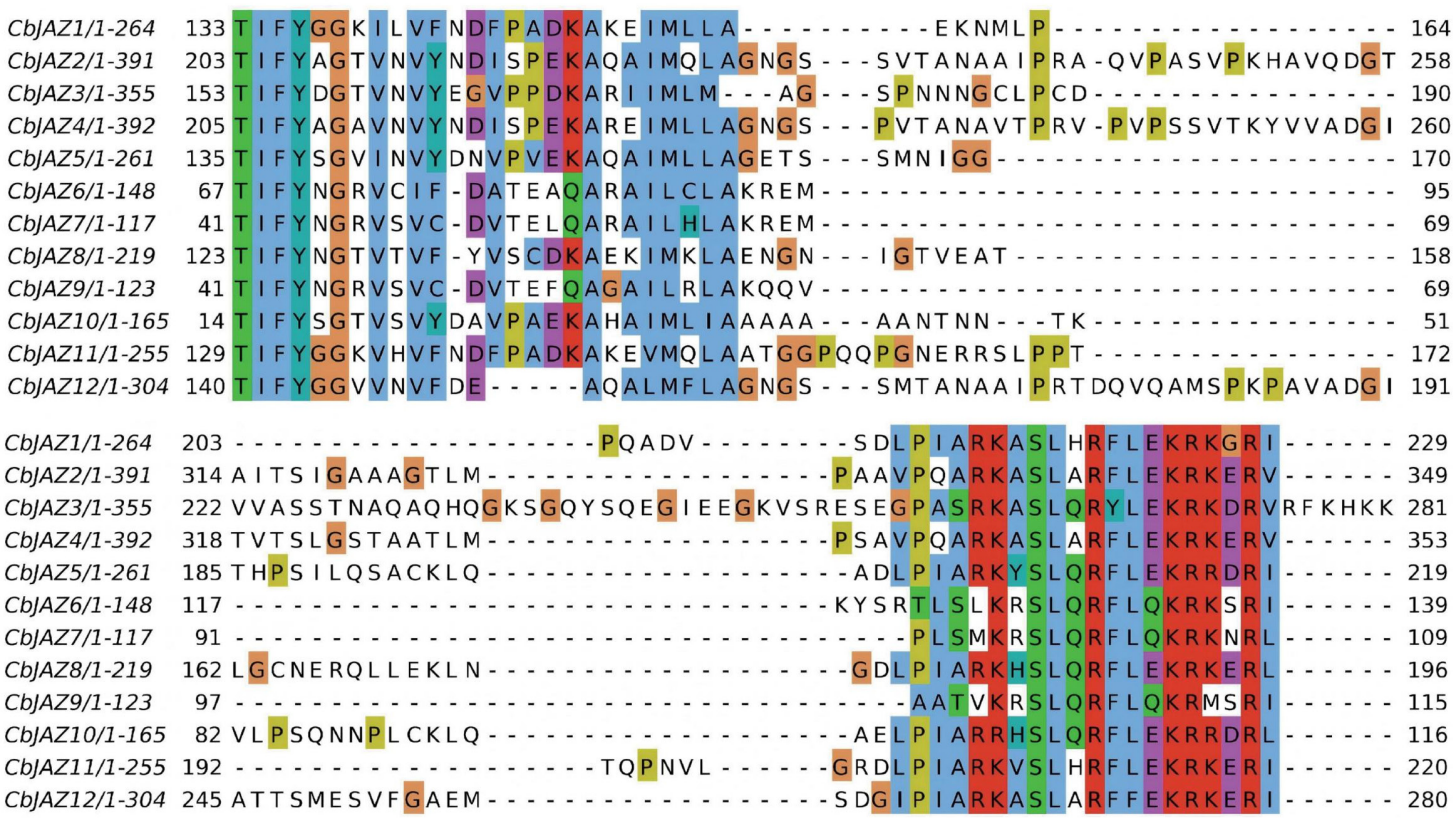
Sequence alignment of CbJAZ family proteins. The upper and lower segments indicate the conserved TIFY and Jas domains, respectively.

**FIGURE 2:**
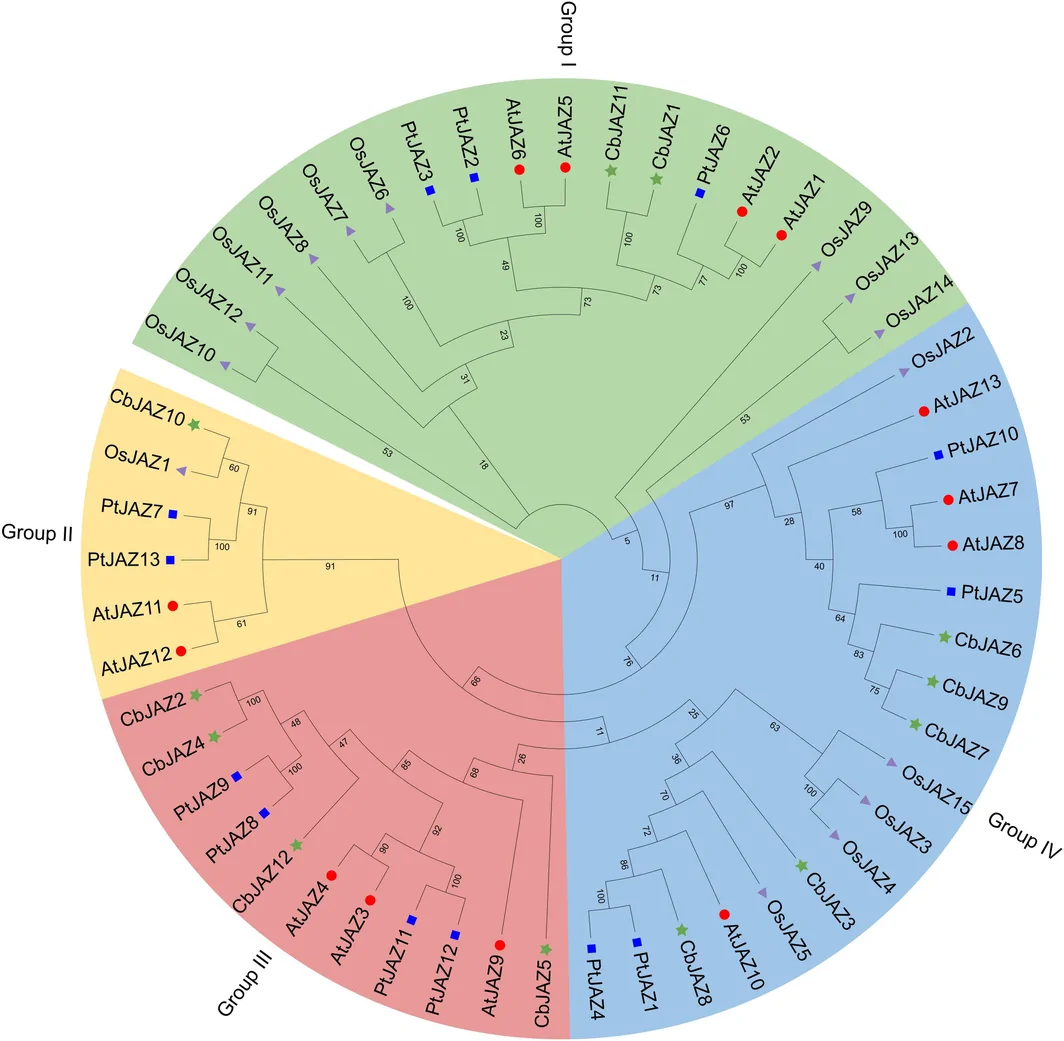
Neighbor‐joining phylogenetic tree showing the relationships among JAZ proteins from *C. burmannii* , *Arabidopsis*, *P. trichocarpa* , and *O. sativa* .

### 3.3 Gene Structure and Conserved Domains Characterization

As shown in Figure 3, the 12 CbJAZ proteins were characterized by their conserved motif composition, domain arrangement, and exon–intron organization. A MEME‐based search identified 10 conserved motifs, among which Motif 1 corresponded to the Jas (CCT_2) domain and Motif 2 to the TIFY domain; both were present in all 12 CbJAZ proteins. Members of the same phylogenetic subgroup exhibited comparable motif compositions, suggesting functional conservation. The remaining motif information is provided in Table S2. These findings were further corroborated by NCBI CDD analysis, which confirmed the presence of both TIFY and CCT_2 domains in all CbJAZ proteins, consistent with the motif distribution. Alignment of genomic and CDSs showed that exon numbers ranged from 3 to 8 across *CbJAZ* genes. Moreover, the exon–intron structures were largely congruent with the phylogenetic subgroups, reinforcing the credibility of the evolutionary classification. We further examined the intron phase within the Jas motif coding region. A conserved Phase 2 Jas intron was identified in eight of the 12 *CbJAZ* genes, positioned at the codon specifying the conserved Arg20. The remaining four genes lack this canonical Jas intron (Figure S1).

**FIGURE 3:**
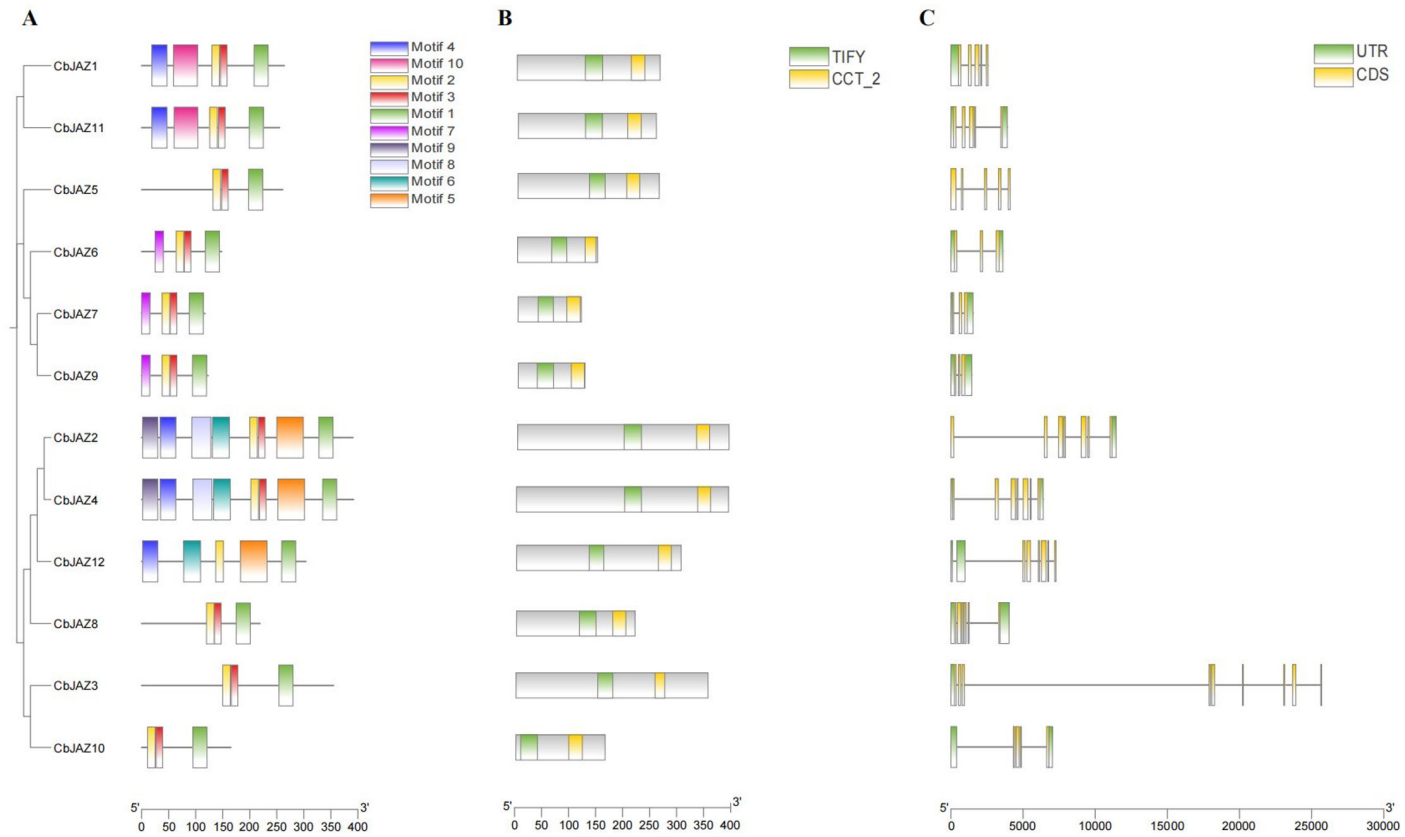
Overview of conserved motifs, domain architecture, and gene structures of CbJAZ proteins from *C. burmannii* . (A) Distribution of conserved motifs identified by the MEME program across CbJAZ proteins. (B) Conserved domain organization of the CbJAZ proteins as determined by NCBI CDD. (C) Exon–intron structures of *CbJAZ* genes, including untranslated regions (UTRs), coding sequences (CDSs), and introns.

### 3.4 Chromosome Distribution and Gene Duplication

The *CbJAZ* members were distributed across seven chromosomes out of the 12 in the *C. burmannii* genome, showing an uneven pattern, namely, Chr01, Chr02, Chr03, Chr04, Chr07, Chr10, and Chr12 (Figure 4A). Gene family expansion is largely driven by gene duplication events. To elucidate the duplication patterns underlying the *CbJAZ* family, we performed comparative synteny analyses between *C. burmannii* and two dicots ( *P. trichocarpa* and *A. thaliana* ) as well as one monocot ( *O. sativa* ). Figure 4B shows that seven segmental duplication pairs were identified within the 12 *CbJAZ* genes, implying that segmental duplication was a major force driving the expansion of the *JAZ* family in *C. burmannii* . Homologous *JAZ* gene pairs were detected between *C. burmannii* and each of the other three species (Figure 4C), with counts of 14 ( *P. trichocarpa* ), 12 ( *A. thaliana* ), and 9 ( *O. sativa* ). Among these, 8, 8, and 4 syntenic linkages were established between *CbJAZ* genes and their orthologs in the three species, respectively. The closer genomic structural conservation between *C. burmannii* and poplar is likely reflected by the higher number of syntenic *JAZ* gene pairs, suggesting that these two woody species may have shared similar evolutionary constraints on *JAZ* genes.

**FIGURE 4:**
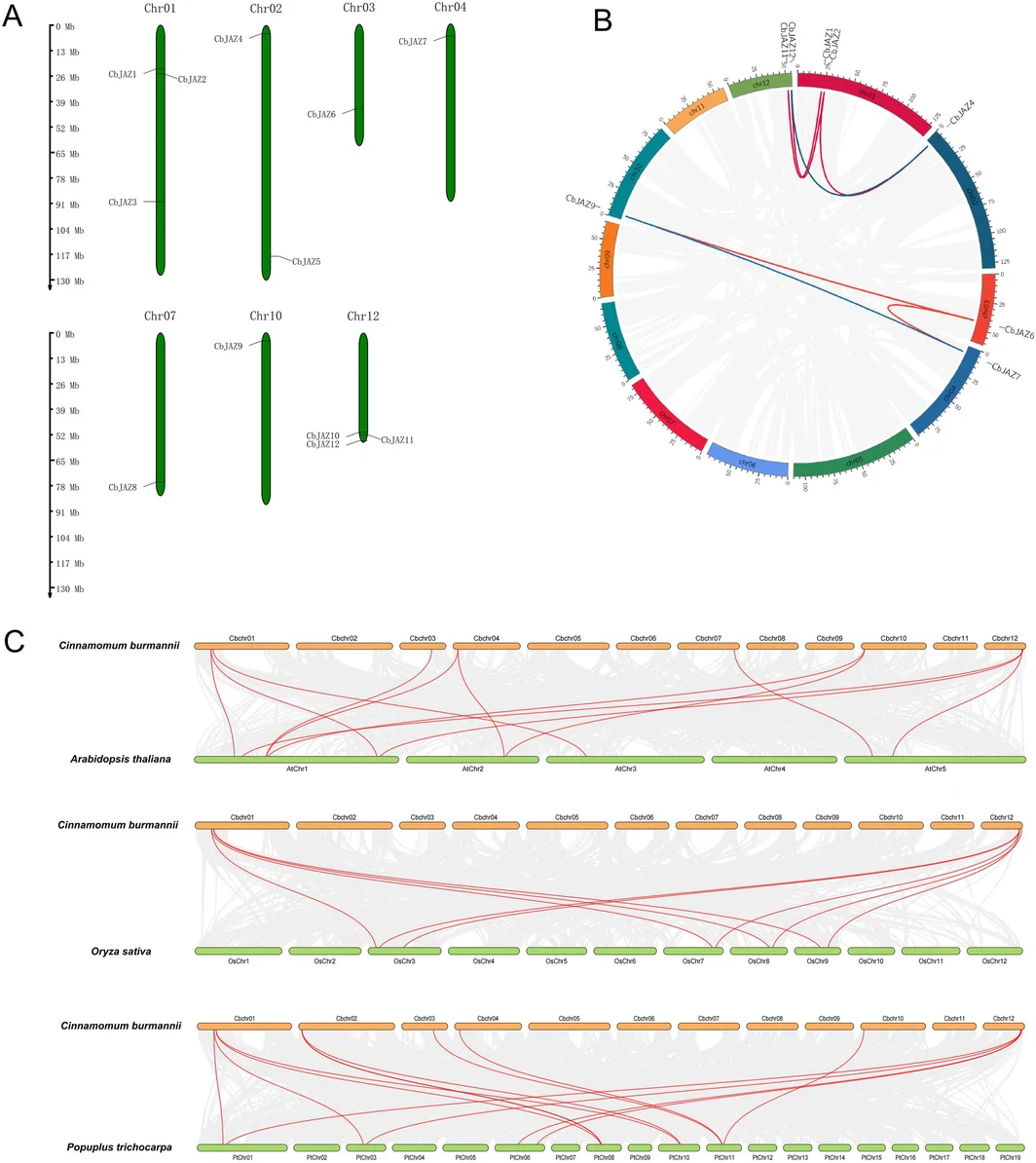
Chromosome distribution and gene collinear analysis. (A) Chromosomal distribution of the 12 *CbJAZ* genes on *C. burmannii* chromosomes. (B) Intra‐genomic collinearity analysis of the *CbJAZ* gene family. Gray lines represent conserved syntenic blocks, whereas colored lines indicate segmentally duplicated *CbJAZ* gene pairs. (C) Syntenic relationships of JAZ genes between *C. burmannii* and three reference species ( *A. thaliana* , *O. sativa* , and *P. trichocarpa* ). The gray lines depict background syntenic blocks shared between the *C. burmannii* genome and each of the other genomes. The red lines identify orthologous *CbJAZ* gene pairs across species.

### 3.5 Identification of Promoter *cis*‐Regulatory Elements

Promoter *cis*‐regulatory elements are informative for predicting the functional divergence and tissue‐specific expression patterns of gene family members. A diverse array of *cis*‐acting elements was identified in the promoters of all *CbJAZ* genes, with highly uneven distribution across family members. Based on their predicted functions, these *cis*‐elements fell into four categories: light‐responsive, hormone‐responsive, stress‐responsive, and those involved in growth and development (Figure 5A). Among these, light‐responsive motifs were the most prevalent, with the G‐box, TCT, and GT1 motifs being the major types. Notably, the G‐box appeared most frequently and was detected in every *CbJAZ* gene. Hormone‐responsive motifs ranked second in abundance, including MeJA‐responsive elements (CGTCA/TGACG), ABA‐responsive elements (ABRE), GA‐responsive elements (P‐box, TATC‐box, GARE), auxin‐responsive elements (TGA, AuxRR‐core, AuxRE), and SA‐responsive elements (TCA). Notably, ABRE motifs were present in all CbJAZ promoters. Stress‐related *cis*‐elements identified included ARE (anaerobic induction), MBS (drought‐associated MYB binding site), LTR (low‐temperature), and TC‐rich repeats (defense/stress). Developmental and growth‐related motifs comprised CAT‐box (meristem), O_2_ site (zein regulation), circadian control element, RY‐element (seed), and GCN4_motif (endosperm). Overall, these findings suggest that *CbJAZ* genes may be regulated by diverse biological processes, including light signaling, hormone transduction, stress adaptation, and developmental regulation (Figure 5B).

**FIGURE 5:**
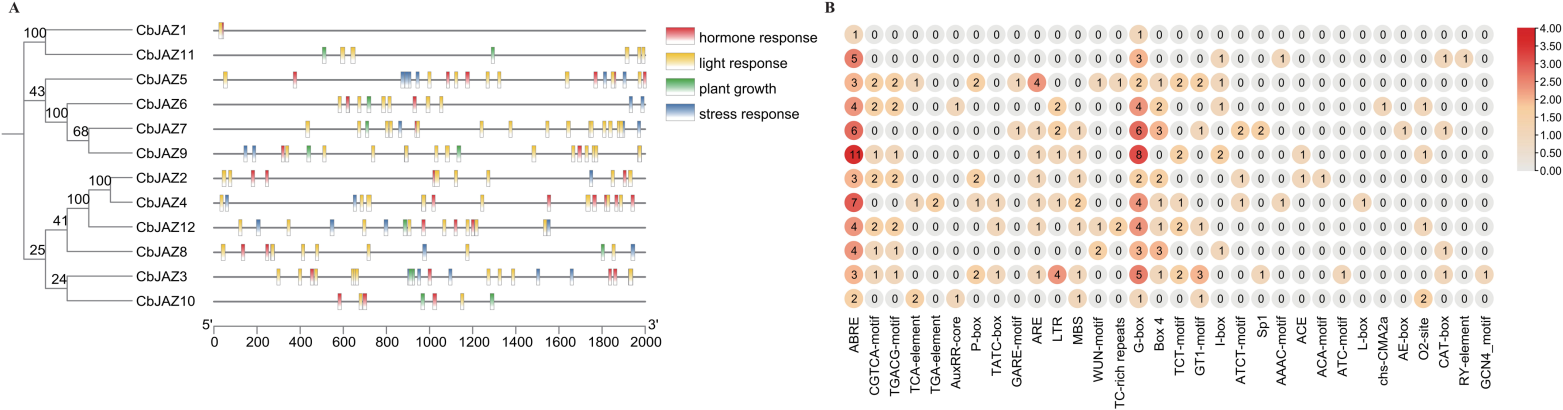
Promoter *cis*‐element analysis of CbJAZ genes. (A) Motif distribution across promoters. (B) Classification of *cis*‐motifs by putative function: light, hormone, stress, and development.

### 3.6 Expression Pattern Analysis of *CbJAZ* Genes in Different Tissues

To gain deeper insights into the spatial expression profiles of *CbJAZ* genes in *C. burmannii* , we quantified the transcript levels of all 12 family members in eight tissues, including roots, stems, fruits, flowers, and four sequential leaf developmental stages, by qRT‐PCR (Figure 6). Pronounced tissue specificity was observed for *CbJAZ* genes, with their expression profiles falling into three major categories based on transcript abundance. The first group consists of *CbJAZ1*, *CbJAZ6*, *CbJAZ7*, *CbJAZ8*, *CbJAZ9*, and *CbJAZ11* and shows preferential expression in reproductive organs. All six genes reach their highest transcript abundance in flowers, with moderately high expression also detected in fruits, whereas their levels in vegetative tissues remain relatively low. The second group comprises *CbJAZ2* and *CbJAZ10*, which display constitutive expression across all examined tissues. Notably, both genes maintain stably high transcript levels throughout leaf development and represent the core *JAZ* members functioning in leaf physiological processes. The third group contains the remaining four genes, *CbJAZ3*, *CbJAZ4*, *CbJAZ5*, and *CbJAZ12*, which exhibit low basal expression in most tissues and are only transiently upregulated at specific leaf developmental stages. Taken together, the divergent tissue expression patterns indicate functional differentiation within the *CbJAZ* family. The predominance of most members in reproductive organs and leaves implies their crucial involvement in regulating reproductive development and leaf‐associated biological processes such as terpenoid biosynthesis in *C. burmannii* .

**FIGURE 6:**
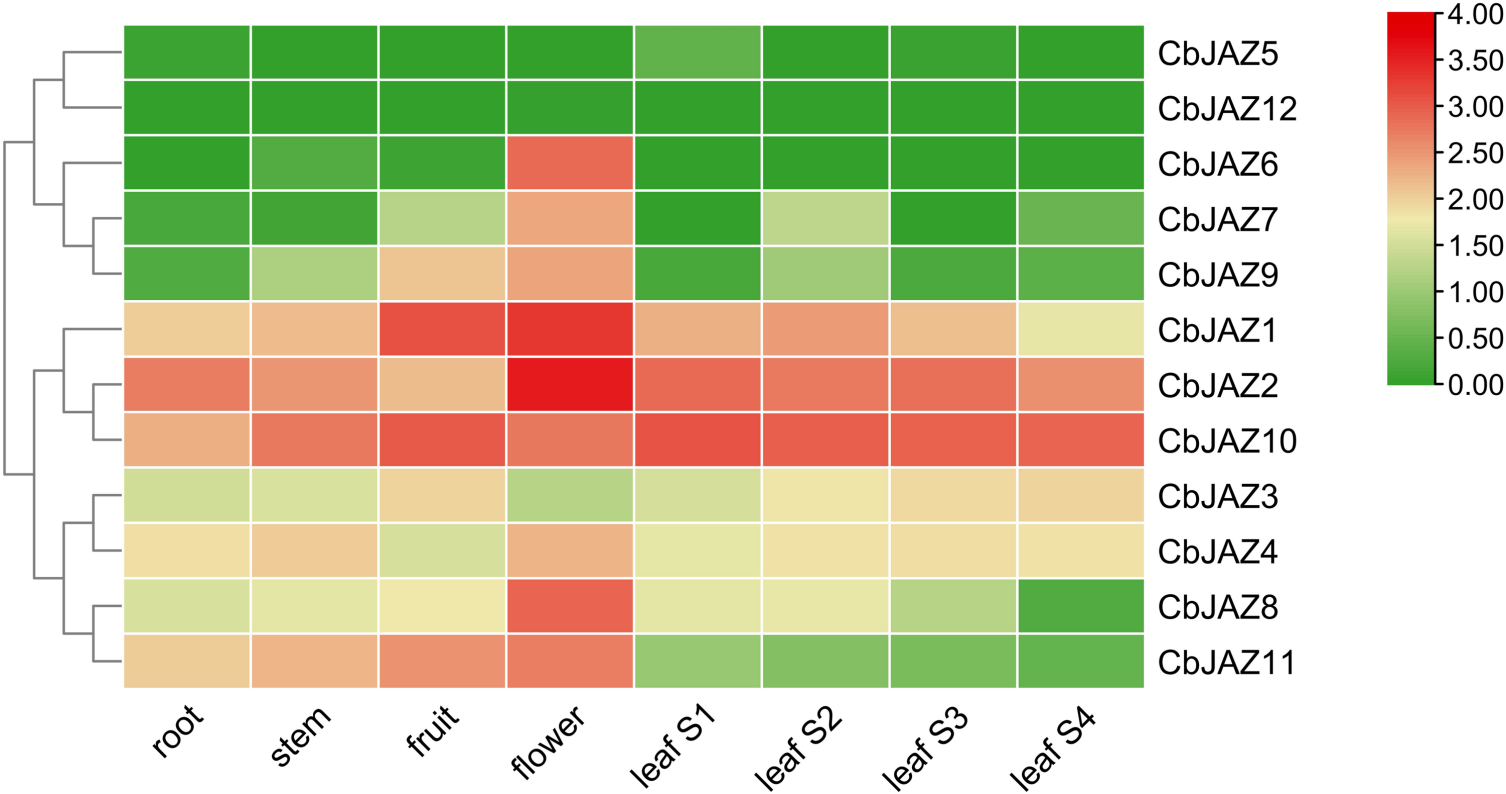
Tissue‐specific expression patterns of *CbJAZ* genes in *C. burmannii* . Transcript levels were examined in roots, stems, fruits, flowers, and leaves at four distinct developmental stages.

### 3.7 Expression Pattern Analysis of *CbJAZ* Genes in Response to Hormone and Drought Stress

We performed qRT‐PCR to examine the transcriptional responses of *CbJAZ* genes to MeJA, ABA, and drought stress. These treatments were selected because MeJA directly activates JA signaling, whereas ABA and drought are key abiotic stresses known to interact with JA pathways. The expression pattern varied under different treatments, whereas most genes shared similar response trends under the same treatment despite differences in induction fold and response timing. Under MeJA treatment, nine *CbJAZ* genes showed upregulated expression to varying degrees (Figure 7A). Among them, the expression levels of *CbJAZ1*, *CbJAZ4*, *CbJAZ6*, *CbJAZ7*, *CbJAZ8*, *CbJAZ9*, and *CbJAZ10* increased first and then decreased with prolonged treatment time. Notably, *CbJAZ8* and *CbJAZ9* showed the most dramatic induction, with expression levels increased by more than 15‐fold at 6 h after treatment, suggesting their transcriptional regulation in response to MeJA. Under ABA treatment, the expression pattern was largely opposite to that observed under MeJA (Figure 7B). Several genes, including *CbJAZ1*, *CbJAZ7*, *CbJAZ8*, *CbJAZ9*, and *CbJAZ10*, were significantly repressed at the early stage of ABA treatment and gradually recovered to basal levels at later time points. Collectively, these results indicate that *CbJAZ* genes are responsive to both JA and ABA signaling and may be involved in the regulation of both pathways. Under PEG 6000‐simulated drought stress, only *CbJAZ1* was significantly induced at 6 h (Figure 7C). The transcript levels of *CbJAZ2*, *CbJAZ4*, *CbJAZ7*, *CbJAZ8*, *CbJAZ9*, *CbJAZ10*, *CbJAZ11*, and *CbJAZ12* were suppressed by drought stress to varying degrees. Notably, *CbJAZ8* and *CbJAZ9* showed significant responses to all three treatments, making them candidate genes for further investigation linking JA, ABA, and drought responses.

**FIGURE 7:**
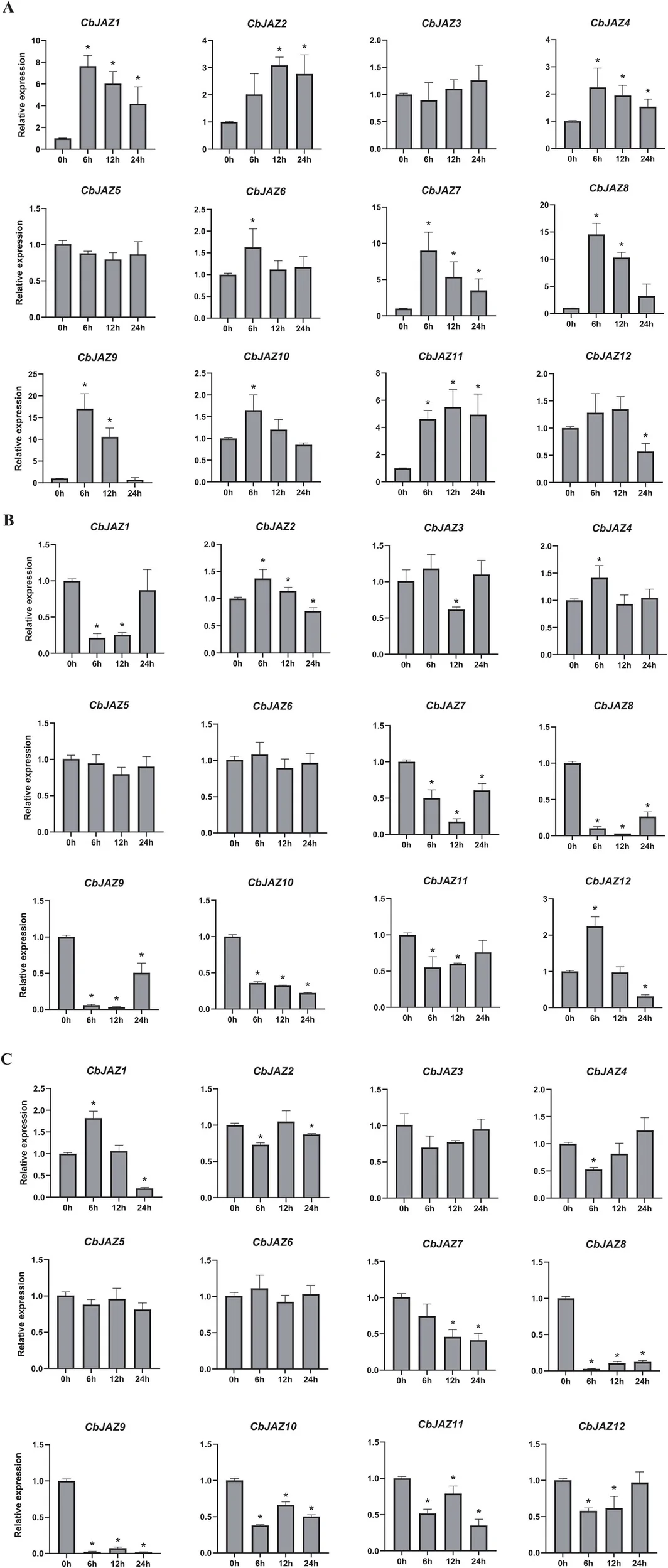
Expression dynamics of CbJAZ genes under hormones and drought stress. (A) Expression after MeJA treatment. (B) Expression after ABA treatment. (C) Expression under drought stress treatments. Significance relative to the 0 h control is indicated by asterisks (*p* < 0.05).

### 3.8 Subcellular Localization of CbJAZ8 and CbJAZ9 Proteins

WoLF PSORT predicted nuclear localization for CbJAZ8 and CbJAZ9 (Table 1). To experimentally verify this prediction, we constructed CbJAZ8‐GFP and CbJAZ9‐GFP fusion vectors and transiently expressed the fusion proteins in tobacco epidermal cells, given that transcription of both genes was markedly induced by phytohormone treatments. Confocal laser scanning microscopy revealed that GFP fluorescence derived from CbJAZ8‐GFP and CbJAZ9‐GFP overlapped perfectly with the mCherry nuclear marker signal, demonstrating that CbJAZ8 and CbJAZ9 are nuclear‐localized proteins (Figure 8), which is consistent with the in silico prediction.

**FIGURE 8:**
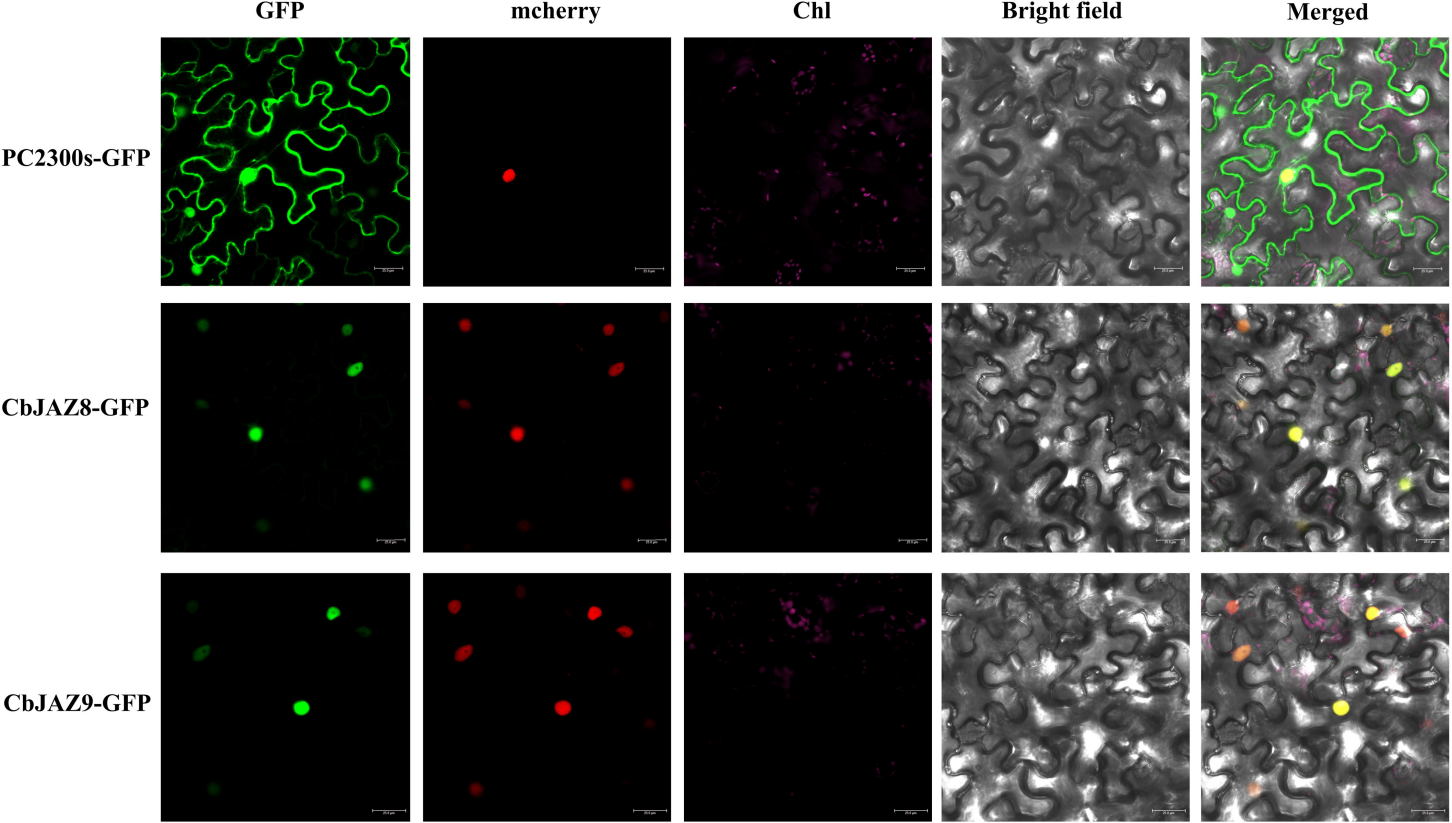
Subcellular localization of the CbJAZ8 and CbJAZ9 proteins. Representative images of GFP signal, mCherry nuclear marker, chlorophyll autofluorescence, bright field, and their merged overlay are presented from left to right. Scale bars = 25 μm.

### 3.9 Correlation Analysis Between *CbJAZ*s Expression and Essential Oil Content

To explore the relationship between JA signaling and essential oil accumulation, we measured essential oil content from the same leaf samples at 0, 6, 12, and 24 h after MeJA treatment and performed Pearson correlation analysis. The essential oil content increased from 0 to 6 h, reached its peak at 6 h, and then gradually decreased at 12 and 24 h (Figure 9A). Correlation analysis revealed that three *CbJAZ* genes, namely, *CbJAZ7* (*r* = 0.98, *p* = 0.02), *CbJAZ8* (*r* = 0.96, *p* = 0.04), and *CbJAZ9* (*r* = 0.97, *p* = 0.03) showed significant correlations with essential oil content (*p* < 0.05) (Figure 9B). Among these, *CbJAZ9* exhibited the highest correlation coefficient. These results suggest that these *CbJAZ* genes may be closely associated with MeJA‐induced essential oil accumulation in *C. burmannii* leaves.

**FIGURE 9:**
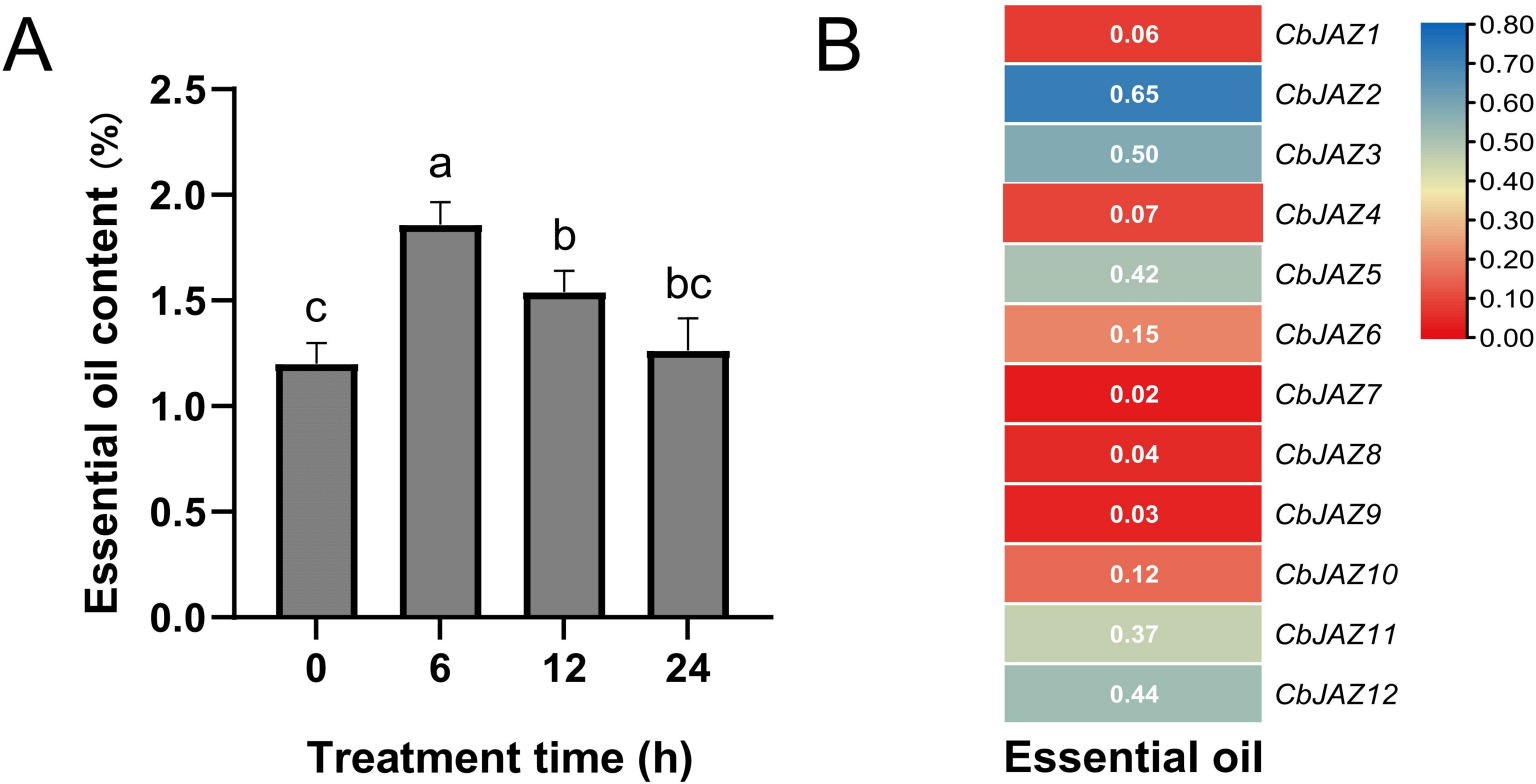
Correlation analysis. (A) The content of essential oil in the *C. burmannii* leaves after MeJA treatment. (B) Heatmap of Pearson correlation *p*‐values between the expression levels of *CbJAZ* genes and essential oil content. Red blocks indicate significant correlations (*p* < 0.05); blue blocks indicate nonsignificant correlations.

## 4 Discussion

### 4.1 Overview of the *CbJAZ* Gene Family

The *JAZ* gene family is widely distributed across plants and plays a central role in JA signal transduction. In this study, 12 *CbJAZ* genes were identified in the *C. burmannii* genome, a number comparable to those reported in *Arabidopsis* and tomato (13 and 12 members) (Liu et al. 2021; Chini et al. 2017), but substantially fewer than those in species with expanded *JAZ* families such as potato (26) (Pu et al. 2025). This moderate family size is notable: Although some woody perennials such as *Rosa hybrida* (32) (Zhu et al. 2025) and *Pinus tabuliformis* (53) (Zhang et al. 2022) have undergone substantial JAZ expansion, *C. burmannii* appears to have maintained a relatively compact JAZ repertoire. All 12 CbJAZ proteins contain the conserved TIFY and Jas/CCT_2 domains, indicating that they possess the structural basis for canonical JA signaling, including dimerization and interaction with COI1 and MYC2.

Phylogenetic analysis classified the 12 CbJAZ proteins into four subgroups (Groups I–IV), consistent with the grouping of *Arabidopsis* JAZ proteins. Notably, the CbJAZ proteins clustered more closely with JAZ proteins from *P. trichocarpa* than with those from *Arabidopsis* or rice, reflecting the closer evolutionary relationship between these two woody perennials. Notably, many CbJAZ members derived from segmental duplication were assigned to different phylogenetic subgroups, echoing observations in *Hevea brasiliensis* where segmentally duplicated *JAZ* genes tend to separate into distinct clusters, whereas tandem duplicates cluster together (Chao et al. 2019). The closer phylogenetic affinity between *C. burmannii* and poplar JAZ proteins likely reflects evolutionary constraints shared by woody perennials, where JA signaling is intertwined with long‐term developmental processes such as secondary growth and stress memory. In poplar, *PagJAZ5* has been shown to regulate cambium activity and promote xylem differentiation during wood formation (Hu et al. 2024), providing direct evidence for the involvement of JAZ repressors in wood development in perennial woody species. Such constraints may have shaped the JAZ family in a lineage‐specific manner, consistent with observations that stress‐responsive gene families often exhibit divergent regulatory patterns between annuals and perennials (Neale and Kremer 2011). Based on this phylogenetic proximity, we tentatively inferred the potential functions of several *CbJAZ* members. For example, *PtJAZ3*, implicated in abiotic stress responses, groups with *CbJAZ1* and *CbJAZ11*, suggesting that these two proteins may also participate in stress regulation (Yang et al. 2023). Similarly, the functionally characterized salt‐tolerance regulators such as *OsJAZ9* cluster with *CbJAZ1* and *CbJAZ11* in Group I, indicating their possible involvement in salt stress responses (Wu et al. 2015).

Gene structure analysis revealed that exon numbers range from 3 to 8, and members within each subgroup share highly similar exon–intron organizations and motif compositions, reinforcing the reliability of the phylogenetic classification. The strict conservation of the TIFY and Jas/CCT_2 domains, together with the observed structural and phylogenetic characteristics, strongly suggests that the CbJAZ family retains the classical functions of JAZ repressors and forms the molecular foundation of JA signal transduction in *C. burmannii* .

The presence of a conserved Phase 2 Jas intron in eight *CbJAZ* genes suggests that alternative splicing may produce truncated JAZ proteins lacking the C‐terminal X5PY sequence. In *Arabidopsis*, such truncated proteins act as dominant repressors of JA signaling because they cannot interact with COI1 but still bind to MYC2 (Chung et al. 2010). This suggests that this post‐transcriptional regulatory mechanism is likely conserved in *C. burmannii* . The loss of this intron in the other four genes may reflect functional divergence within the family.

### 4.2 Gene Duplication and Evolutionary Expansion of the *CbJAZ* Family

Gene duplication events, including segmental and tandem duplications, serve as primary drivers for the expansion of gene families. In this study, collinearity analysis identified seven segmental duplication events among the 12 *CbJAZ* genes (Figure 4B), whereas no tandem duplication events were detected. This indicates that segmental duplication, rather than localized tandem repeats, has played the predominant role in shaping the *CbJAZ* family. Within the same genus, the absence of tandem duplication was also observed in *Cinnamomum camphora* , where no tandemly duplicated gene pairs were detected among the 11 *JAZ* family members (Zhang, Liu, et al. 2024). Similarly, in *Manihot esculenta* , no tandem clusters were detected among JAZ, PPD, and ZML genes (Zheng et al. 2022). However, this pattern is not universal. Five new *JAZ* genes in wheat were found to have originated from tandem duplication after its divergence from other species (Zhai et al. 2024). The high duplication‐to‐gene ratio (7 pairs among only 12 members) suggests that duplicated *CbJAZ* genes have been preferentially retained, likely contributing to the fine‐tuning of JA signaling across different tissues or environmental conditions.

Inter‐species synteny analysis further revealed that *C. burmannii* shared more homologous JAZ gene pairs with the woody dicot *P. trichocarpa* (14 pairs) than with *A. thaliana* (12 pairs) or the monocot *O. sativa* (9 pairs). However, the number of syntenic blocks with poplar (8) was equal to that with *Arabidopsis* (8), both doubling that with rice (4). This indicates that although gene pair conservation is higher between the two woody species, the overall genomic organization of *JAZ* loci is equally well conserved across dicots. Such syntenic stability contrasts with maize, where limited synteny with Arabidopsis has been attributed to their long divergence time (Han and Luthe 2021). Notably, *CbJAZ2* and *CbJAZ11* possessed orthologous syntenic blocks in all three compared species, paralleling the deep conservation of *MsJAZ8* and *MsJAZ17* in alfalfa (Yan et al. 2024). This suggests that *CbJAZ2* and *CbJAZ11* are deeply conserved across angiosperms and may constitute core components of the JA signaling machinery, maintained since the divergence of major angiosperm lineages.

In summary, the *CbJAZ* family has been shaped primarily by segmental duplication, with duplicated members preferentially retained. The deep syntenic conservation of *CbJAZ2* and *CbJAZ11* highlights their potential as core JA signaling components, providing a framework for future functional dissection of individual *CbJAZ* members in hormone signaling and stress adaptation.

### 4.3 *Cis*‐Element Analysis and Hormonal Regulation of *CbJAZ* Genes

Promoter analysis revealed that the upstream regions of *CbJAZ* harbor diverse *cis*‐regulatory motifs involved in light, hormonal, stress, and developmental signaling (Figure 5). Among these, the G‐box was the most frequent motif and was detected in every *CbJAZ* gene. The G‐box is a primary binding site for bHLH transcription factors, including light‐responsive PIFs and JA‐responsive MYCs, suggesting it may integrate both light and JA signals. Other hormone‐responsive motifs included MeJA‐responsive CGTCA/TGACG motifs, ABRE, and elements responsive to gibberellin, auxin, and salicylic acid. Notably, ABRE elements were present in the promoters of all 12 *CbJAZ* genes, and similar promoter architectures have been reported in wheat (Wang et al. 2017) and *Artemisia argyi* (Gong et al. 2025). Collectively, *CbJAZ* genes are broadly equipped with hormone‐ and stress‐responsive regulatory motifs, implying their potential involvement in multiple signaling pathways. Our qRT‐PCR results further validated this prediction, showing that *CbJAZ* genes exhibited distinct expression patterns under MeJA, ABA, and drought treatments. Globally, the majority of *CbJAZ* genes were transcriptionally activated by MeJA but repressed by ABA and drought stress, forming a reciprocal response pattern that supports the widely recognized antagonistic crosstalk between JA and ABA signaling pathways.

In line with findings in numerous plant species ranging from *Arabidopsis* to woody trees, MeJA treatment induced the expression of most *CbJAZ* genes in a transient manner. This MeJA‐responsiveness is consistent with observations across diverse taxa: In wheat, the majority of *TaJAZ* genes were upregulated following MeJA treatment (Wang et al. 2017); in sweet potato, most *IbJAZ* members were also induced by MeJA, with several genes showing over 40‐fold induction (Huang et al. 2021); and in sugarcane, all seven *ScJAZ* genes were rapidly upregulated by MeJA, peaking at 3 h before declining (Liu et al. 2017). This MeJA responsiveness extends to nonvascular plants; in the model liverwort *Marchantia polymorpha* , the single MpJAZ repressor controls the jasmonate pathway, and the COI1‐JAZ co‐receptor complex is responsive to jasmonate treatment (Monte et al. 2019; Chini et al. 2021). This rapid induction followed by attenuation represents a classic negative feedback loop inherent to JA signaling. In the canonical JA pathway, bioactive JA triggers the degradation of JAZ repressors to release downstream transcription factors; meanwhile, JAZ genes themselves are transcriptionally activated by released MYC2 and other JA‐responsive factors, and the newly synthesized JAZ proteins in turn dampen JA signaling to prevent excessive defense activation and growth inhibition (Bowman et al. 2017; Thines et al. 2007). The conserved expression kinetics observed in *C. burmannii* suggest that this core negative feedback mechanism is retained in the Lauraceae family. Notably, *CbJAZ8* and *CbJAZ9* displayed the strongest and earliest induction (over 15‐fold at 6 h) among all family members, indicating that these two genes may act as major negative regulators in the JA signaling cascade of *C. burmannii* , playing a predominant role in resetting JA homeostasis after stimulus perception.

In contrast, exogenous ABA significantly suppressed multiple *CbJAZ* genes at early time points. This appears seemingly contradictory to the ubiquitous ABRE motifs in all CbJAZ promoters, since ABRE is generally regarded as a positive *cis*‐element. However, several mechanisms may explain this discrepancy: ABRE can function as either an activation or repression motif depending on the recruited bZIP factors; ABA may regulate *CbJAZ* expression indirectly by modulating MYC transcription factors or other JA pathway components; and *cis*‐element regulatory output is context‐dependent, determined by copy number, position, and flanking sequences. Similar ABA‐mediated repression of JAZ genes has been reported in sweet potato (Huang et al. 2021) and *Brassica napus* (Wang et al. 2022; Chini et al. 2021), indicating that ABA antagonism of JA signaling at the JAZ transcriptional level is a widespread regulatory mechanism. The reciprocal regulation of *CbJAZ8* and *CbJAZ9*, combined with the enrichment of both ABA‐ and MeJA‐responsive elements in their promoters, positions these two proteins as candidate genes potentially involved in JA‐ABA crosstalk.

Under PEG‐simulated drought stress, most *CbJAZ* genes showed sustained downregulation, consistent with the ABA response pattern and physiologically reasonable given that drought triggers endogenous ABA accumulation. This drought‐repressive pattern contrasts with JAZ upregulation observed in annual herbs such as *Brassica rapa* (Liang et al. 2025) and *Mentha canadensis* (Xu et al. 2021). We propose this reflects distinct stress adaptation strategies between woody perennials and herbaceous annuals. Long‐lived evergreen trees like *C. burmannii* may favor a resource‐conservation strategy: prioritizing ABA‐mediated drought acclimation while downregulating *JAZ* genes to reduce metabolic cost and balance growth with survival. In contrast, annual herbs with short life cycles tend to fully activate both JA and ABA pathways to maximize stress resistance and ensure reproduction before senescence. *CbJAZ1* was transiently upregulated at the early stage of drought, suggesting functional differentiation among family members.

Among all *CbJAZ* genes, *CbJAZ8* and *CbJAZ9* stood out for their robust responses to all three treatments: strongly induced by MeJA and markedly repressed by ABA and drought. Combined with their confirmed nuclear localization, we propose *CbJAZ8* and *CbJAZ9* as candidate genes that warrant further functional validation, converging JA, ABA, and drought pathways, potentially modulating JA signaling output in response to environmental cues to orchestrate trade‐offs between growth, defense, and stress tolerance. It should be emphasized that this hypothesis is based solely on expression profiling data; the underlying molecular mechanisms await validation through protein interaction assays and genetic functional studies.

### 4.4 Tissue‐Specific Expression of *CbJAZ* Genes

The tissue expression profiles revealed pronounced spatial differentiation among *CbJAZ* genes, which can be roughly divided into three categories: reproductive organ‐preferential, constitutive, and low‐abundance with stage‐specific peaks. This divergent expression pattern indicates extensive functional differentiation within the *CbJAZ* family, with different members likely participating in distinct physiological processes across organs and developmental stages.

Notably, most *CbJAZ* genes (including *CbJAZ1*, *CbJAZ6*, *CbJAZ7*, *CbJAZ8*, and *CbJAZ9*) exhibited the highest transcript levels in flowers, with moderately high expression also detected in fruits (*CbJAZ1*, *CbJAZ7*, and *CbJAZ9*) (Figure 6), consistent with previous reports on *JAZ* gene expression in reproductive tissues (Chini et al. 2018; Serrano‐Bueno et al. 2022). In aromatic plants, flowers and fruits are not only core organs for reproductive development but also major sites for the biosynthesis of terpenoid compounds that mediate pollinator attraction and defense against floral pathogens (Mostafa et al. 2022). Extensive studies in medicinal and aromatic plants have established a conserved regulatory module in which JAZ proteins repress terpenoid biosynthesis by physically interacting with MYC2, thereby inhibiting the expression of terpene synthase genes (Cao et al. 2024; Wang et al. 2024; Zhu et al. 2022). The preferential expression of *CbJAZ7*, *CbJAZ8*, and *CbJAZ9* in flowers, together with their significant correlations with MeJA‐induced essential oil accumulation in leaves, suggests that these genes may be associated with JA‐responsive metabolic processes. A similar positive correlation between JAZ expression and terpenoid accumulation has been reported in *G. biloba* , where *GbJAZ01*, *GbJAZ02*, and *GbJAZ07* showed significant positive correlations with terpene trilactone content (He et al. 2022).

This flower‐ and fruit‐biased expression pattern is not unique to *C. burmannii* but has been observed across a wide range of plant species, especially in medicinal and aromatic plants with rich terpenoid metabolism. A similar trend was found in the congeneric *C. camphora* , where multiple JAZ members (*CcJAZ3*, *CcJAZ4*, *CcJAZ6*, and *CcJAZ11*) were also predominantly expressed in flowers, and *CcJAZ4*, *CcJAZ5*, *CcJAZ7*, and *CcJAZ9* showed preferential expression during seed development (Zhang, Liu, et al. 2024), suggesting that the involvement of *JAZ* genes in floral secondary metabolism may be a conserved feature in *Cinnamomum* species. Similarly, flower‐dominant JAZ expression has been reported in *Panax notoginseng*, where JAZ I subgroup genes were highly expressed in flowers (Yang et al. 2025). Likewise, in *Toona ciliata* , a woody timber species, *TciJAZ* genes were also predominantly expressed in flowers and tender stems (Song et al. 2022). In contrast, some species display different tissue‐specific patterns, such as *Artemisia annua* , where *AaJAZ5* showed the highest expression in leaves (Xia et al. 2018), and *S. miltiorrhiza* , where multiple *SmJAZ* genes displayed constitutive expression across roots, stems, and leaves (Li et al. 2021).

In addition to reproductive organs, leaves are the primary tissue for essential oil extraction in *C. burmannii* , and essential oil content varies dynamically during leaf development. Notably, several *CbJAZ* genes showed stage‐specific expression during leaf development. For instance, *CbJAZ2* and *CbJAZ10* showed constitutively high expression throughout all leaf stages, suggesting that they may serve as potential regulators to maintain homeostatic control of JA signaling during leaf growth. In contrast, *CbJAZ5* was specifically highly expressed in young leaves (S1), whereas *CbJAZ7*, *CbJAZ8*, and *CbJAZ9* peaked at the mature leaf stage (S2), matching the general trend that terpenoid biosynthesis gradually increases as leaves mature. These stage‐specific *CbJAZ* members may fine‐tune JA‐dependent terpenoid production in coordination with leaf developmental programs, which may help coordinate essential oil accumulation with the physiological state and defense requirements of leaves at different ages.

Collectively, the tissue‐specific expression patterns of *CbJAZ* genes provide important spatial and temporal clues for their functional divergence. The reproductive organ‐biased and leaf development‐associated expression profiles not only lay a foundation for dissecting the developmental regulation of JA signaling in this aromatic tree but also pinpoint priority candidate genes for future research on JA‐mediated essential oil metabolic engineering in *C. burmannii* . Nevertheless, the potential roles of these candidate genes in JA‐mediated essential oil metabolism remain to be experimentally validated through protein interaction assays and functional characterization.

## 5 Conclusions

Through genome‐wide identification, we first obtained 12 *CbJAZ* genes from *C. burmannii* , all containing the conserved TIFY and Jas domains. The expansion of the *CbJAZ* family was driven primarily by segmental duplication, which generated seven duplicated gene pairs among the 12 members, whereas no tandem duplication events were observed. Cross‐species synteny revealed that *CbJAZ2* and *CbJAZ11* are deeply conserved across dicots and monocots. Promoter *cis*‐element analysis revealed an abundance of hormone‐ and stress‐related motifs, whereas expression profiling showed that most *CbJAZ* genes responded positively to MeJA but negatively to ABA and drought. Among them, *CbJAZ8* and *CbJAZ9* emerged as candidate genes potentially involved in JA‐ABA antagonistic crosstalk. Tissue‐specific expression further revealed flower‐ and fruit‐biased expression patterns of most *CbJAZ* members, with additional stage‐specific expression during leaf development, suggesting their potential involvement in both reproductive development and leaf‐associated physiological processes. These findings provide a foundation for future functional dissection of *CbJAZ* genes and provide candidate gene resources for subsequent studies on stress response mechanisms and essential oil metabolic regulation in *C. burmannii* . It should be noted that our conclusions are primarily based on expression profiling and correlation analysis. Direct functional evidence for the involvement of *CbJAZ8* and *CbJAZ9* in JA‐ABA‐drought crosstalk remains to be established in future studies.

## Author Contributions

W.L.: conceptualization, methodology, investigation, formal analysis, data curation, writing original draft. X.C. and Y.L.: investigation, data curation. X.L.: writing review and editing. Z.C.: conceptualization, supervision, writing review and editing. All authors read and approved the final manuscript.

## Funding

This research was funded by the Technology Program from Forestry Administration of Guangdong Province (Grant Numbers 2026KJCX001 and 2019KJCX029).

## Conflicts of Interest

The authors declare no conflicts of interest.

## Supporting information


**Table S1:** Sequences of primers used in qRT‐PCR.
**Table S2:** The structural features of motifs.
**Figure S1:** Exon–intron structures of CbJAZ genes.

## Data Availability

Data are contained within the article and the Supporting Information.
