# Supplementary material for: Genome‐Wide Identification and Stress‐Responsive Expression of the JAZ Gene Family in Cinnamomum burmannii
Source: Plant Direct. 2026 Oct 21;10(10):e70195. doi: 10.1002/pld3.70195 (PMC13686813; doi:10.1002/pld3.70195)
Supplement: Supplementary file 1 [file PLD3-10-e70195-s001.docx]

| **Table S1 Sequences of primers used in qRT-PCR.** | | |
| --- | --- | --- |
| **Gene name** | **Forward primer sequence (5′ → 3′)** | **Reverse primer sequence (5′ → 3′)** |
| *CbRPS15* | GCAGCCGAAGAGGAGAACA | GGCTTCCGCTTCAAACCAC |
| *CbJAZ1* | AGTACCTGAAGGAGAAGGGGAC | GCACGAGGCGGAAAAAAG |
| *CbJAZ2* | GAGACTGTGGATGGAAGCAAAG | GGCTGAAAAGCAGATGAGGTAA |
| CbJAZ3 | CTTCTCCTCCCTTGCCTGTT | AAGAAGACGAGCCCCCGAT |
| CbJAZ4 | TCAAGGCAGCAACAGAAGAGA | TGATTGGAAGCAGGGAAGG |
| CbJAZ5 | ATGTTGTCGGGGACTACCATTC | TCATGCCATATGAGCTGAGACA |
| *CbJAZ6* | TCGTCCTTGCCATCACTATCTC | GCGGTGGTTCAAGTCATCTCC |
| CbJAZ7 | GAAGATTCAACTGGAAACGAGC | TCGCAAGGTGTAAGATGGCT |
| CbJAZ8 | CGGAAAATGGAAATATCGGA | GGAATAACTGGCAAAAGGAGAA |
| CbJAZ9 | TCTTTGCCTTCTTCCAGCG | TGTTGCTTTGCGAGGCGTA |
| CbJAZ10 | GTCAGCGTCTATGATGCGGT | CAGCAGAACTCACTGCCGTATT |
| CbJAZ11 | CAATCCCACACAGCCCAAC | AAGAAGTCAACCCGAGCCAC |
| CbJAZ12 | CAATGGAGTCTGTTTTTGGAGC | AGGGGGAGGCATTTGTTATC |

| **Table S2 The structural features of motifs.** | | | | | |
| --- | --- | --- | --- | --- | --- |
| **Name** | **E-value** | **Sites** | **Width** | **Amino acid sequence** | **Logo** |
| Motif 1 | 5.5e-123 | 12 | 27 | DLPIARKASLQRFLEKRKERITSKSPY |  |
| Motif 2 | 3.6e-089 | 12 | 15 | QLTIFYNGTVNVYDD |  |
| Motif 3 | 5.3e-035 | 11 | 13 | PPEKARAIMLLAG |  |
| Motif 4 | 7.8e-020 | 5 | 29 | QKPSFSNKVSALSHFLKFKGAFEERPLKM |  |
| Motif 5 | 1.4e-013 | 3 | 50 | KHAVADGINGNQSQASSPCSGLSSPISVTSHAGAHSGGASCNTDDLIAIK |  |
| Motif 6 | 7.1e-008 | 3 | 32 | NHSFSVAMTNPFFKIHGGVGGPSVTTNSVKQQ |  |
| Motif 7 | 8.2e-008 | 3 | 16 | MGRGCNLDLCLLPAGI |  |
| Motif 8 | 2.7e-007 | 2 | 36 | QKTFNLDRQGAHFAMQAYPFNSVDGSHCSQEIRTFP |  |
| Motif 9 | 1.5e-004 | 2 | 29 | RDFLGLNIKEPMALVKEETVDGGKDQVFM |  |
| Motif 10 | 1.6e-004 | 2 | 45 | FIPPTTMNLLPKIEISGEGPZRKENGSDRNIKSMDFFPPRVGFGP |  |

**Figure S1. Exon-intron structures of *CbJAZ* genes.**
